# Imaging Atomic Scale Dynamics on III–V Nanowire Surfaces During Electrical Operation

**DOI:** 10.1038/s41598-017-13007-w

**Published:** 2017-10-06

**Authors:** J. L. Webb, J. Knutsson, M. Hjort, S. R. McKibbin, S. Lehmann, C. Thelander, K. A. Dick, R. Timm, A. Mikkelsen

**Affiliations:** 1 Division of Synchrotron Radiation Research, Lund University, Lund, Sweden; 2 Division of Solid State Physics, Lund University, Lund, Sweden

## Abstract

As semiconductor electronics keep shrinking, functionality depends on individual atomic scale surface and interface features that may change as voltages are applied. In this work we demonstrate a novel device platform that allows scanning tunneling microscopy (STM) imaging with atomic scale resolution across a device simultaneously with full electrical operation. The platform presents a significant step forward as it allows STM to be performed everywhere on the device surface and high temperature processing in reactive gases of the complete device. We demonstrate the new method through proof of principle measurements on both InAs and GaAs nanowire devices with variable biases up to 4 V. On InAs nanowires we observe a surprising removal of atomic defects and smoothing of the surface morphology under applied bias, in contrast to the expected increase in defects and electromigration-related failure. As we use only standard fabrication and scanning instrumentation our concept is widely applicable and opens up the possibility of fundamental investigations of device surface reliability as well as new electronic functionality based on restructuring during operation.

## Introduction

Significant changes in performance during operation have been observed in electronic devices for many years, although the precise microscopic structural origin often remains unclear^1^. As component dimensions progress to the atomic scale, the geometry and electrical response of single atom defects will significantly influence performance^2,3^. Especially important are surface properties as even small changes of structural features (during electrical operation) can alter device function^4–6^ due to the large surface to bulk ratio of nanoscale structures, novel 2D materials^7,8^ or single molecular^9^ based devices.

Techniques that can relate surface geometry and electronic structure to electrical performance are thus highly important, of which scanning tunnelling microscopy (STM) uniquely allows imaging of both surface geometry and electronic structure down to individual atoms and as such already play a prominent role in the strive towards atomic scale semiconductor electronics^2,3,9–11^. In particular, the surfaces of III–V semiconductors are a proven base for STM studies of fundamental atomic scale semiconductor processes^2,12–18^. For larger (micrometre sized) surfaces considerable structural rearrangements have been observed by STM under applied bias^19–21^. However, the high resolution capabilities of STM have not been extended to the case of electrically active nanostructured semiconductor surfaces.

In realising such studies, III–V nanowires have a number of important features. They can be tailored into a wide variety of atomically precise axial and radial heterostructures with well defined composition, crystal structure and morphology^22,23^. This makes them highly useful both as components in future (opto)electronics^24–26^ as well as versatile systems for fundamental studies of semiconductor nanoscale confined surfaces. The 1D geometry allows local control of the voltage and thermal profiles along the wire, which have proven difficult for larger thin films^19^. Importantly, it has been demonstrated that STM/scanning tunnelling spectroscopy(STS) studies to the atomic scale are possible on a wide variety of nanowires^4,27,28^ giving both structural arrangements and electronic properties such as bandgap and band alignment.

The most common device for studies of nanowire device functionality has a lateral design whereby the wires are deposited on insulating SiO_2_ and subsequently top contacted by electron beam lithographically (EBL) and thermal deposition of metal^29–31^. This structure can be passivated, oxidised and top gated through lithographic patterning and can be probed at lower resolution by alternative scanning probe techniques^32,33^. It has been used in our previous work utilising combined AFM/STM scanning to perform measurements on the device surface^31^ However, in trying to extend directly on this work to obtain true atomic resolution on controlled nanowire surfaces we have identified several serious shortcomings for atomic scale STM. Firstly, on this standard device only the metal contacts and the nanowire itself are conducting, limiting STM scanning to these areas. The tip can often move onto the insulating SiO_2_ where it is rendered permanently useless by surface crash. Secondly, the height of the contacts precludes scanning near the important nanowire-contact region. Thirdly and most seriously, the strong mechanical grip of overlaid contact electrodes can cause the nanowire to fracture during heating and cooling procedures above 300 C^34^. This has been observed with the device electrically isolated, ruling out electromigration-related failure.

A prerequisite for any successful STM study to the atomic scale is the presence of a well defined crystalline surface with a limited (controllable) number of structural defects. To achieve this surface treatments above 400–500 C in reactive gases such as atomic hydrogen^4,35^ must be possible on the device platform. In contrast the common nanowire device has an oxidised surface due to transport at ambient air conditions which in reality precludes atomic resolved studies using STM (or AFM). Previous studies have thus been limited to significantly lower imaging resolution. Importantly such uncontrolled surface oxide conditions also makes it impossible to determine the role of individual specific types of defects. Having the oxide free crystalline surface as a starting point allows for the introduction and study of specific defects (through additional overgrowth or gas dosing for example) while applying bias and studying surface properties.

In this work we demonstrate via a series of proof of principle measurements on III–V nanowire devices that we can achieve atomic scale resolution imaging of InAs and GaAs surfaces by ultra high vacuum (UHV) STM with simultaneous electrical operation of the nanowire device. We demonstrate four major capabilities of our method: atomic resolution during operation, atomic level surface control, nanoscale imaging in combination with large voltage variations and finally high resolution electrical potential mapping. We show that atomic defects on the InAs surfaces move, reorder and vanish under applied bias and with larger nanoscale changes in surface structure while local variation in surface potential are extracted from topographic images across a crystal stacking fault.

## Results

### The new device platform

**Figure 1:**
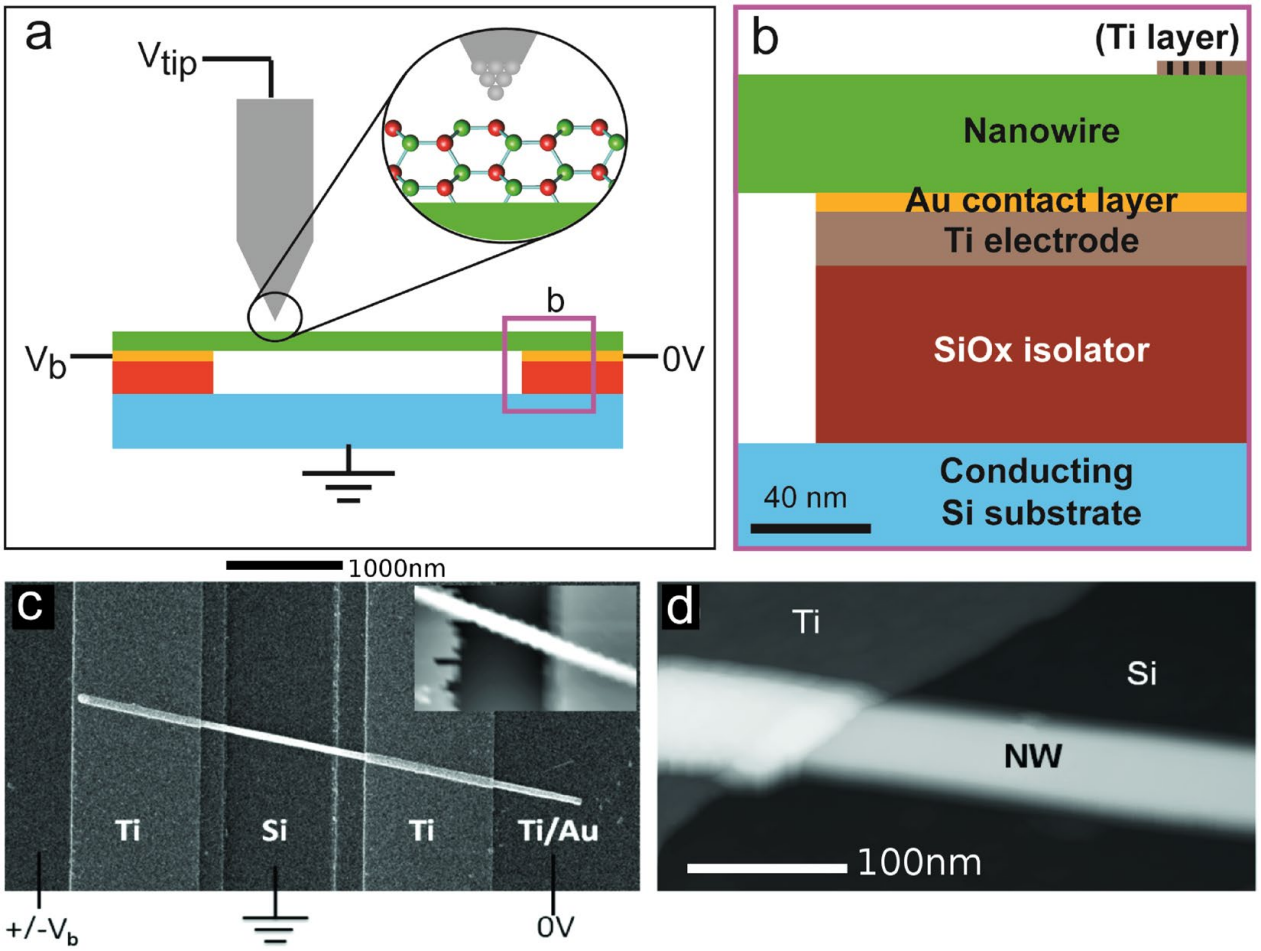
Nanowire device for atomically resolved STM. (**a**) Schematic of the STM device experiment. The nanowire (in green) has been cleaned and atomic resolution is possible as illustrated in the zoom-in. V_*b*_ is defined as the bias applied across the nanowire. V_*tip*_ the bias on the STM tip with respect to the ground. The nanowire contacts can be independently grounded which prevents electrical shorting and defines a reference ground level (and reference height for the STM tip). (**b**) Profile of the nanowire-contact area in (**a**). The nanowire crosses a conducting Si trench contacted by Ti/Au electrodes and separated from the conducting Si substrate by thermally deposited SiO (or sputter grown SiO_2_). The Si substrate can either be grounded or used as a back gate. The nanowire can be held in position by an optional thin Ti ‘holding’ layer deposited on top of it parallel to the trench, but significantly away from the trench edge. (**c**) A top down SEM image of the completed device, showing the nanowire above a 1 *μ*m wide Si trench and contact electrodes (labelled). Inset: Fast scan overview STM image of Ti/Au contacts and nanowire, the image is 3 × 2 *μ*m. (**d**) Higher resolution STM image of a nanowire device, showing the Ti/Au contact, nanowire above the Si substrate. All regions of the device can be scanned by the STM tip at tip bias of −1.5 V in constant current mode at setpoint 60 pA.

We have developed a new device design suitable for STM imaging as shown in Fig. 1, consisting of a semiconductor nanowire suspended on top of two conducting electrodes that can be independently biased and which are electrically isolated from the substrate. Unlike the >100 nm SiO_*x*_ thermally grown insulating oxide, the Si substrate has only a thin native oxide layer sufficiently conductive to allow STM scanning on it. A key point is the design of the layers of the contacts as shown in Fig. 1b), where the Si*O*_*x*_ insulating layer isolating the metal electrodes from the conductive substrate is only found underneath the contact and not visible to the STM tip. This permits scanning anywhere on the sample surface whilst electrically insulating the nanowires from ground and allowing a bias to be applied only across them. Placing the nanowires on top of the electrodes also reduces strain in the nanowire during heating, preventing fracture of the wires due to surface cleaning. To prevent problems with alloying between contacts and the nanowire while retaining an Ohmic, low resistance junction, a thin Au capping layer (<5 nm) was used, as opposed to thicker Au contacts used in more standard designs^36^. The detailed fabrication and experimental layout is described in Methods. It retains the standard processing techniques and materials (Si, Ti, Au) to provide a realistic alternative that can be easily fabricated and keep many of the properties of the conventional lateral design.

A top-down SEM image of the new device structure after complete processing can be seen in Fig. 1c) which was transported in air to a UHV Omicron STM system. Oxide-free, crystalline nanowire surfaces were obtained by atomic hydrogen cleaning at 400–500 C (see Methods) inside an adjacent preparatory chamber under UHV, with immediate transfer into the STM chamber. Large scale STM imaging as seen in the inset of Fig. 1c) was used to locate a given device nanowire (the full location procedure is outlined in ‘Methods’). We could scan across the nanowire despite the 90–100 nm height difference between the Si substrate and the top of the nanowire. All areas of the device could be scanned without degradation of the STM tip. Figure 1d shows an STM image of part of a nanowire contacted by the Ti/Au electrode and suspended above the Si substrate (black), used for positioning of the STM at any specific distance from the contacts or any other relevant (hetero)structure along a nanowire.

### Atomically resolved imaging during device operation

**Figure 2:**
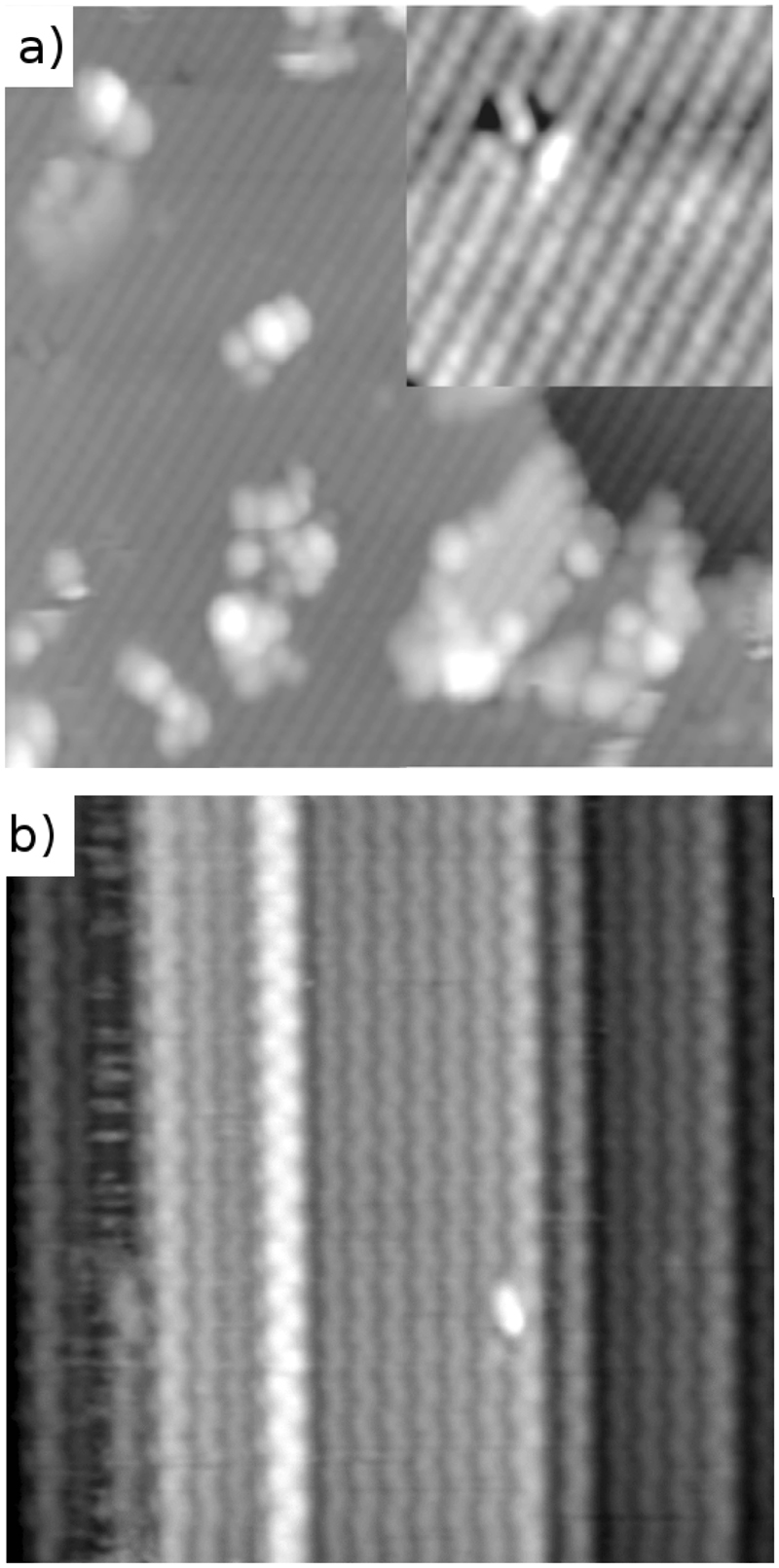
STM imaging with atomic resolution on both InAs and GaAs nanowire devices. (**a**) 20 × 20 nm^2^ STM image of the (110) surface of a GaAs nanowire with zincblende structure. The figure inset more clearly shows the atomic resolution from the top layer As atoms on the crystalline surface with the individual As atoms of the surface (bright) and a defect consisting of several As vacancies (dark). The image is recorded with V_*b*_ = −3 V applied across the nanowire. (**b**) 20 × 20 nm STM image of a InAs $\mathrm{\{11}\bar{2}\mathrm{0\}}$ facet showing the atomic resolved zig-zag pattern observed on the surface of a wurtzite InAs nanowire device. In this specific session the surface was imaged for about 3 hours under varying bias conditions. Here V_*tip*_ = −2 V in constant current mode at setpoint 90 pA.

As a proof-of-principle of our method for samples with many different structural features, we imaged a GaAs nanowire surface that had a mixture of surface steps, free crystalline surface and defects (largely remaining oxide that can be removed by further cleaning cycles^27^). In Fig. 2a) we show a STM image of this surface while there is simultaneously a V_*b*_ = −3 V bias along the nanowire. This demonstrates that true atomically resolved imaging (even in the presence of steps and defects) is possible with a large voltage drops over the nanowire device. To show that high quality measurements are possible across different materials and crystal structures we show an atomically resolved image from a wurtzite InAs nanowire in the device configuration in Fig. 2b. This is not a trivial observation, as we will now demonstrate how applied bias can seriously affect the semiconductor surfaces.

**Figure 3:**
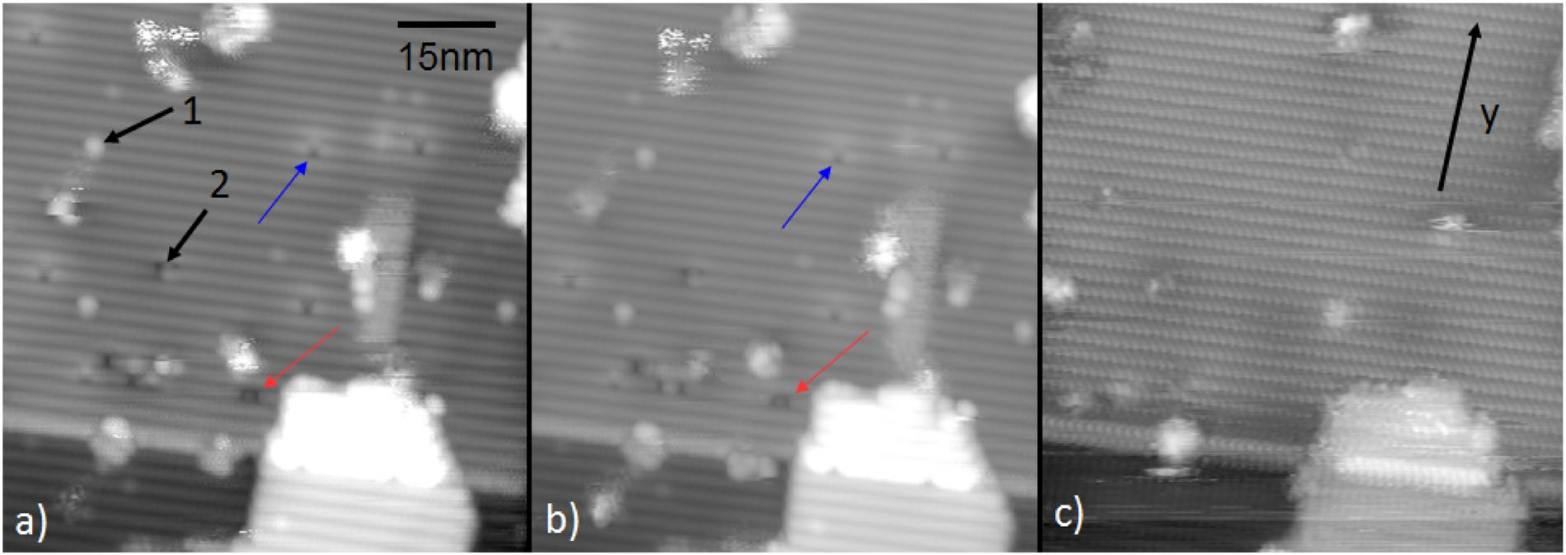
Demonstration of the removal of surface defects by bias application. (**a**–**c**) STM images on an InAs nanowire approximately 200 nm from the biased contact, showing the $\mathrm{\{10}\bar{1}\mathrm{0\}}$ facet with a stacking fault (SF)and numerous point defects and surface features. We probe filled states, with As surface atoms appearing as bright protrusions. All images were taken at V_*b*_ = 0. On (**a**), label 1) is a mobile cluster of surface In atoms, 2) an As vacancy defect. (**b**) Imaging the same position at V_*b*_ = 0 V after 6 scans over one hour at zero applied bias across the nanowire with little tip-induced motion of vacancies. (**c**) Imaging at V_*b*_ = 0 V after V_*b*_ = −2 V had been applied for 10 min, showing substantial motion and elimination of surface point defects. The nanowire growth direction (y, long axis of the nanowire) across which the potential is applied is indicated by an arrow. In all three images both vacancies and adsorbates can be clearly identified (example indicated by blue arrow) even though tip images conditions changed slightly. However, in the third image many defects present previously were no longer visible (example indicated by red arrow). Here V_*tip*_ = −1.5 V in constant current mode at setpoint 80 pA.

We applied the atomically resolved imaging to study surface defect changes and mobility during operation across a uniform InAs nanowire. For these measurements we chose to apply the bias and then image at V_*b*_ = 0 in order to fully and unambiguously distinguish the effect of applied bias from the effect of tip scanning on the surface, which could otherwise potentially generate and rearrange defects independently to the applied bias. Figure 3a) shows the first image in one session of such STM imaging with a high resolution image taken on the $\mathrm{\{10}\bar{1}\mathrm{0\}}$ facet on an InAs nanowire. We observe an unreconstructed flat surface with the same quality as in previous STM studies of grounded InAs nanowires on an InAs substrate^28^. As labelled on the figure, we observe randomly distributed point defects, some resembling As vacancies as previously seen on InAs(110)^37^ as well as crystal stacking faults.

Studying defect dynamics requires imaging stability which we tested by scanning the same area of the surface repeatedly for more than 1 hour with no bias along the nanowire. Figure 3b) shows the same position after this period of continuous scanning at V_*b*_ = 0 V. At V_*b*_ = 0 V the small point defect vacancies and the stacking fault do not alter. Only a few mobile clusters of atoms on the surface moved. After checking that the tip did not significantly influence surface structure, a bias of V_*b*_ = −2 V was applied along the wire for 10 min. The image in Fig. 3c) taken at V_*b*_ = 0 V after this period shows the dramatic effect of the applied bias. The defect density was significantly reduced with many point defects now absent (example indicated by red arrow). The stacking fault remained essentially unaltered, but two vacancies in it were eliminated along with several larger adsorbate features. In all three images atomic resolution is retained, but the tip changes somewhat (as is often the case for extended imaging periods on volatile sample surfaces). This could potentially also render some types of defects difficult to observe and present a general problem. However, both before and after device bias have been applied we can observe some of the same vacancies and adsorbates (example indicated by the blue arrow) while other defects have gone. If the disappearance of some defects were due to a tip change rendering them invisible one would expect that all of these defects would no longer be visible. Since this is not the case we can conclude that the observation of defect changes is possible on III–V surfaces even under structural changes. We address this issue of tip change further through Supplementary Information Figs 2, 3 and 4 where we indicate defects present in scan images before and after bias, an area of surface reconstruction and show that these can still be observed (and observed to have moved or disappeared) even after a tip change. In addition we show the alteration and reconstruction in the direction of current flow of a large scale defect in SI Fig. 5 at atomic resolution with no change in the tip before and after imaging.

We have performed similar experiments to the GaAs(110) surface as seen in Fig. 2. Unlike the observations on InAs we found limited change in defect and surface structure under the same level of applied bias. We attribute this to the higher resistivity and lower current densities in GaAs alongside the generally stronger bonding of the GaAs lattice^38^. This self-cleaning effect upon supplying a voltage through the InAs nanowire is unexpected as prolonged STM scanning and exposure to vacuum leads to the formation of more defects on some III–V surfaces^17^. As significant Joule heating effects can be excluded^39^ and any conceivable temperature rise would lead to formation of more vacancies, we must conclude that it is the electric current or voltage gradient through the wire surface that directly leads to the removal of defects. This will be discussed in more detail below.

### Larger scale reconstruction observed after applying bias

**Figure 4:**
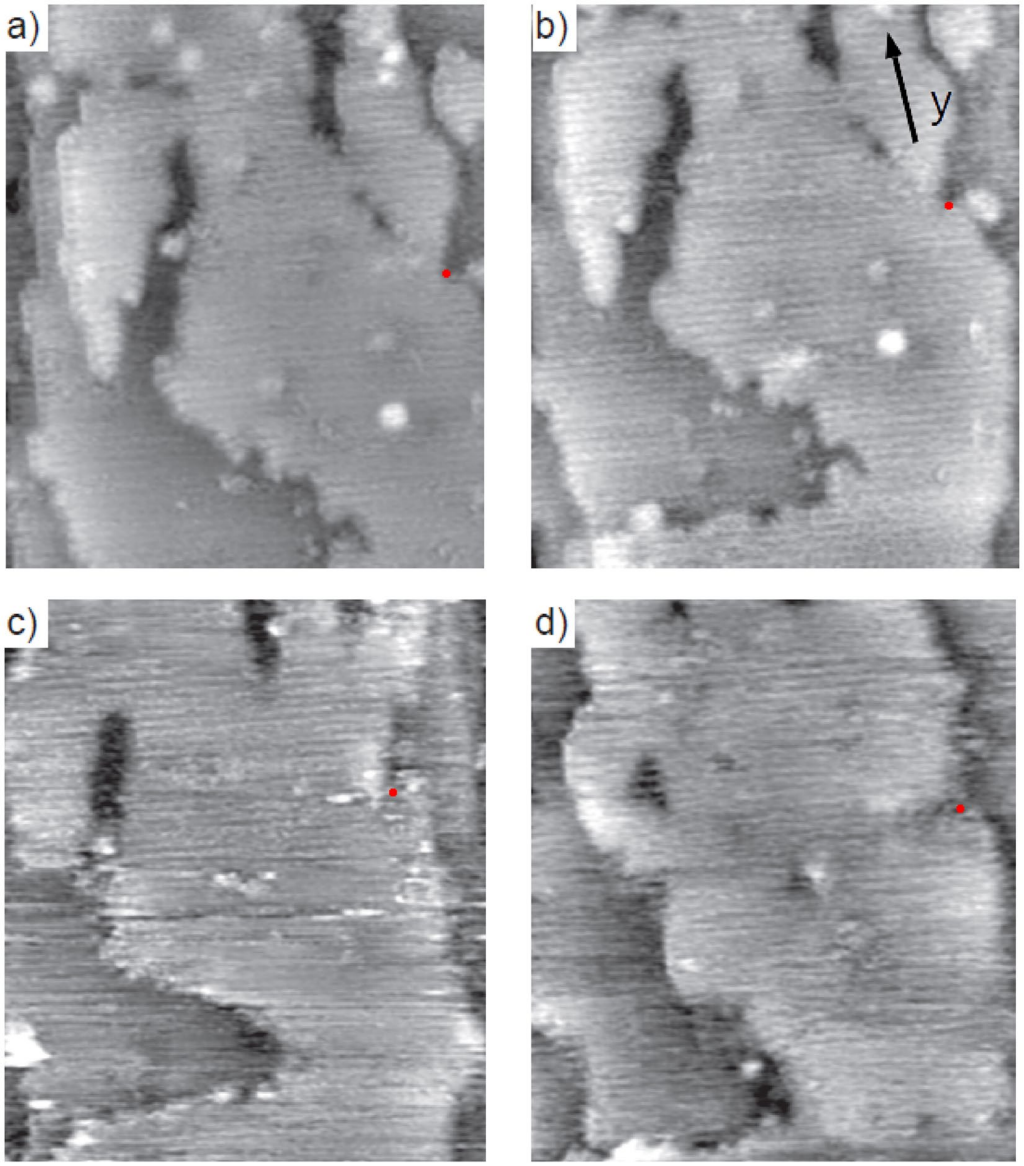
STM imaging of large scale re-arrangements due to applied bias. (**a**–**d**) Shows 40 × 40 nm^2^ STM image scans taken on a wurtzite crystal structure InAs nanowire after successive application of bias ranging from V_*b*_ = 0 to −3 V. (**a**) Taken before application of any bias, (**b**) after application of V_*b*_ = −0.5 V, (**c**) after application of −2 V and d) after −3 V. Images were recorded with V_*b*_ = 0 V. The faint equally spaced lines seen across all images are the atomic rows of the $\mathrm{\{10}\bar{1}\mathrm{0\}}$ surface facet, indicating atomic resolution despite the surface being changed significantly due to the applied bias along the nanowire. An approximate common position on each image is indicated by a red dot and the nanowire growth direction (long axis of the nanowire) across which the potential is applied is indicated by arrow y. Here V_*tip*_ = −1.5 V in constant current mode at setpoint 90 pA.

In order to further explore the opportunities for studies of structural changes and since surface steps can significantly impact nanowire device function (through charging and acting as recombination centres) we investigated larger nanoscale changes in nanowire surface morphology. We therefore investigated larger nanoscale changes in nanowire surface structure. Figure 4 shows a larger scale section of the InAs nanowire surface, imaged after successive application of V_*b*_ ranging from 0 to −3 V for 5–10 min at each bias. We initially observed a surface with a number of larger islands, separated by defects ranging in size from small point defects to the large features separating surface islands of atoms.

Recording many repeated images with no bias applied along the nanowire and we observed some mobile surface clusters that could be picked up and relocated by the tip. This was also the case for biases V_*b*_ < −2 V in Fig. 4a,b where only a few clusters have moved with no evidence of significant changes induced by nanowire bias. After V_*b*_ < −2 V, significant structural change in the surface was observed as seen in Fig. 4c,d. Most prominently the island morphology completely changes and the surface becomes smoother, the larger features between islands changing shape and filling. Eventually a more defect free and stable surface appears as seen in Fig. 4d. At this point, few changes occur even when applying −3 V along the nanowire for 5 min. We speculate that the changes observed are from either direct migration of In/As atoms to fill the defects and regions between islands due to the strong surface electric field under applied bias, or indirectly due to scattering from current flow through the nanowire.

Electromigration of In atoms on the surface of semiconductors is a phenomenon that has been observed previously^40,41^. However, this should be a process that by definition produces more (vacancy) defects in a localised area and severely compromises the crystal structure of the nanowire ultimately ending in its structural failure^42^. In the work by Chao Zhang *et al*. using TEM for example^43^ this occurs close to the electrical contact, at a similar position to our STM scan location. It is thus surprising to see the opposite effect occurring. We account for this via two possible scenarios: firstly, that defect generation (and failure) occurred elsewhere (such as the opposite contact), liberating atoms to move along the surface and fill defects in the scan area or that secondly we were below the critical threshold for this to occur, with the surface rearrangement occurring due to the presence of the strong surface electric field. To exclude in the former case scans were performed moving along the full length of the nanowire observing no such defect accumulation and failure region. Our previous measurements^31^ suggest the clean, oxide free surface of InAs to have higher electrical conductance than the bulk of the nanowire - a change to a defect free, smooth surface should thus produce an increase in conductance.

Some slight image drift was observed, potentially indicative of heating of the surface. However this drift disappeared rapidly (within a single 1–2 min image). Typically for samples heated above room temperature we would observe several hundred nm of drift over such a period. The current (maximum < 1–4 *μ*A) was significantly below that required to heat the nanowire significantly and any heat dissipation from the nanowire should have been rapid due to the good thermal contact with the large metallic electrodes on either end of the nanowire. Recent measurements of the heating of InAs nanowires due to a driving voltage by Menges *et al*.^39^ have shown that heating effects are very small. At the maximum voltages used in the present study a temperature rise of <60 C would be expected. This temperature is too low to explain the observed changes in the surface as Joule heating, in particular since heating at these temperatures of similar surfaces leads to vacancy formation and not removal^17^. Generally vacancy formation has been observed even at room temperature for some III–Vs. However, at the relevant temperatures of this study we only observe As vacancy formation not removal. This conclusion is reached both based on theoretical studies that show how defects are more stable at the surface than in the bulk^44^ and from many experimental observations of vacancy formation of III–V surfaces^13^ and also on nanowires^35^. Works by others also generally find that the STM tip might in some cases generate more vacancies, but not fewer.

An important benchmark for the usefulness of our method towards dynamical studies is that imaging could be resumed and nanowire structural changes observed rapidly after significant changes in the voltage applied across the nanowire, for example within only a few minutes after up to −3 V had been applied to the wire for 10 min and then switched off abruptly. The ability to image immediately after large changes of device voltage contrasts previous studies of electromigration using STM^19^. In this work significant time was needed (>30 min) before the surface became stable enough to perform imaging on due to heating and charging effects. That we are able to measure again so quickly further corroborates the excellent heat and charge dissipation in our setup.

### Probing the effect of surface defects on nanowire conductance

An interesting feature of our setup is the ability to probe the surface potential at any position on the nanowire surface. As we change the applied bias V_*b*_ along the nanowire, the variation in surface potential V_*L*_ alters tunnel current to the tip that in turn will change the z-position Δd due to the response of the STM feedback loop in constant current mode. Using established models for this z-dependence^45^ we can estimate how changes in apparent height variation relate to changes in V_*L*_. This allows direct comparison between the surface electrical conduction at a given point along the nanowire and the surface structure. The concept is based on our ability to vary V_*b*_ during continuous STM imaging of the same area. Assuming that changes in V_*L*_ depend on the local conductance this method further allows an estimate of the effect on conductance of a specific local nanoscale surface feature, which is not possible by standard electrical transport measurements. A detailed explanation of this method is given in Supplementary Information.

**Figure 5:**
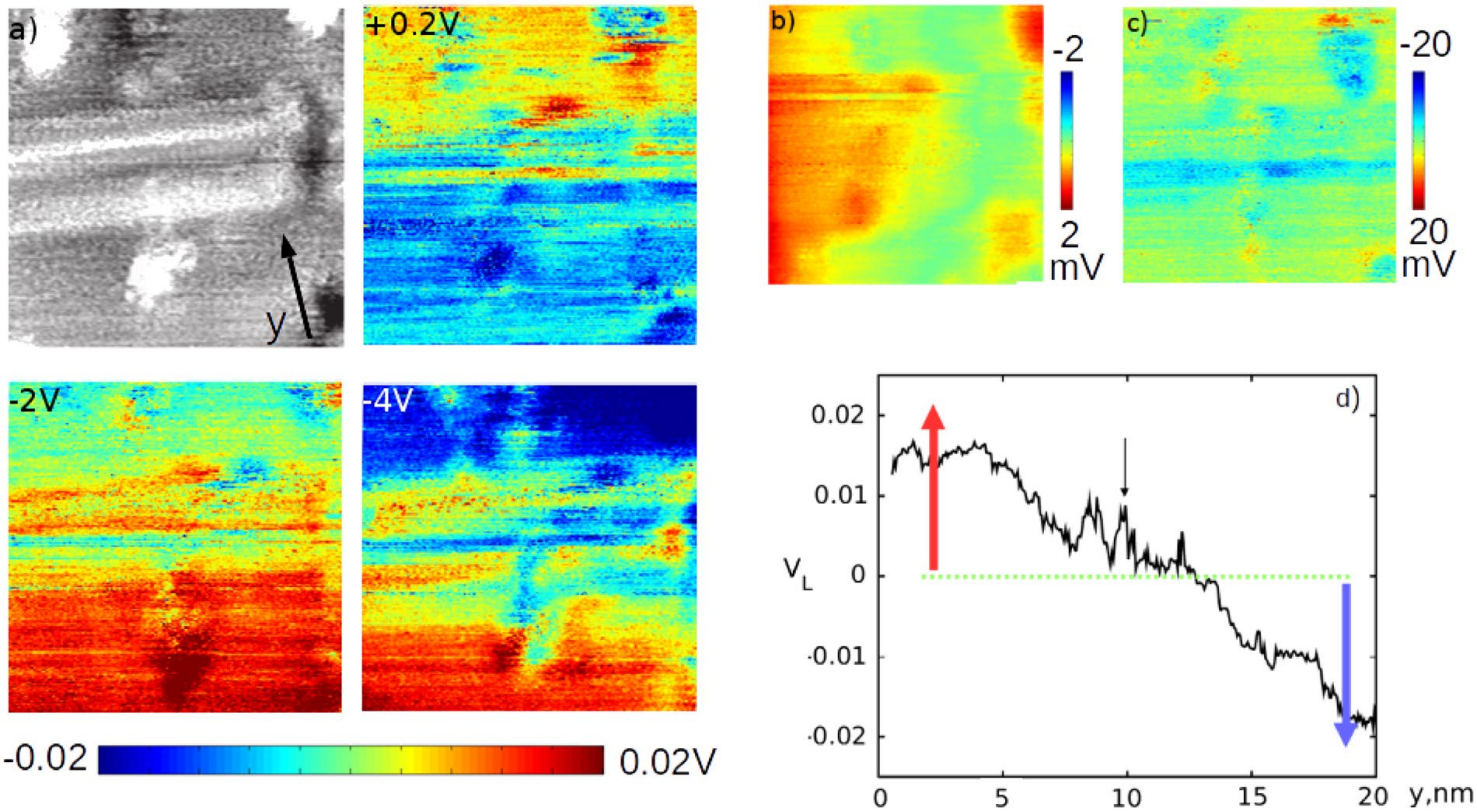
Local nanowire surface potential measured by STM topography. In (**a**) (top left) we show a 20 × 20 nm^2^ STM topography scan image on an InAs nanowire of a stacking fault (SF) at V_*b*_ = 0 V, where y indicates the direction along the length of the nanowire between the contact electrodes. This is shown along with (top right, bottom) the subtracted difference between extrapolated local potential V_*L*_ between V_*b*_ = 0 and V_*b*_ = +0.2, −2 V and −4 V (labelled) with V_*tip*_ = −1.3 V, showing the effect of increasing applied bias. A clear trend was observed of increasing difference in V_*L*_ in y across either side of the stacking fault with increasing V_*b*_. As a control to show this is not a tip artefact we show in (**b**) 20 × 20 nm subtracted difference in extrapolated local potential between scans of this area at V_*tip*_ = −1.7 V and V_*tip*_ = −1.3 V at V_*b*_ = 0 V, with the change of tip bias having effects an order of magnitude below that in (**a**). We also show another control in (**c**) with a 20 × 20 nm subtraction of two consecutive scans at the same V_*b*_ = −1 V, with no gradiation in y of the local potential observed. (**d**) shows a 2D plot of V_*b*_ = −4 V data in (**a**), taking the average calculated V_*L*_ across a 20 nm section. Here the stacking fault position is indicated by an arrow, with the red/blue arrows referring to the colourscheme used in (**a**) to better relate STM topography and V_*L*_.

In order to provide a proof of principle demonstration of this method we study a region with a stacking fault in an InAs nanowire, imaged in Fig. 5a (top left) at V_*b*_ = 0 V. This is the same type of stacking fault as seen in Fig. 3a with atomic resolution. These stacking faults are important common atomic scale structural features in nanowires^46^ that can influence conductance and are clearly visible and in the present case do not change with applied bias along the wire.

A bias dependent difference in absolute STM tip height is observed across the stacking fault where for negative bias the position of the tip was higher below (bottom of the image) as compared to above the stacking fault (top of image) for negative voltages and reversed for positive voltages. This can be seen in Fig. 5 for V_*b*_ = 0.2 V, −2 V and −4 V. We see an increase in the gradient of tip height change from higher (darker blue) to lower (darker red) as V_*b*_ is increased from 0 V to −4 V. This is as expected for a local drop in V_*L*_ across the stacking fault that increases in gradient with V_*b*_. The change in local potential induced by the bias along the nanowire can be seen in the potential drop across the 20 nm section of the InAs nanowire as approximately 0.04 V for V_*b*_ = −4 V. A nanowire feature such as a stacking fault, large defect or heterojunction has a finite electrical resistance and hence can affect the nanowire conductance. Indeed, while the change in V_*L*_ was approximately linear either side of the stacking fault - Fig. 5d - as would be expected from two regions of InAs nanowire with similar resistance, a rather different potential behaviour is seen across the stacking fault.

We rule out several other explanations for the effects observed. Firstly, the effect of changing V_*b*_ (and thus local surface potential) on tip height is somewhat equivalent to changing the tip bias V_*tip*_. At different V_*tip*_, features may appear more or less prominent. These effects should be uniform across the image, being a product of the absolute change in tip height as a result in change in tip potential and should show no dependence in the direction of current flow/applied bias (in y, along the nanowire). Figure 5b shows subtracted height data for images taken at V_*tip*_ = −1.7 V and V_*tip*_ = −1.3 V with the nanowire electrically grounded. No clear difference across the stacking fault was observed, with no dependence on y and a uniform or no height increase across the area of interest. Secondly, a change in the tip configuration could also induce changes between subsequent subtracted images. In Fig. 5c we show 2 consecutive subtracted images taken at the same applied (V_*b*_ = −1 V) and tip bias (−1.5 V) in order to demonstrate that that this cannot account for the effects observed. Thirdly, in addition to the robust effect across the stacking fault we observe localised changes in d(x,y) corresponding to mobile surface features - mobile clusters of surface atoms moved and redeposited by the tip from image to image.

Our method has advantages over alternatives such as scanning tunnelling spectroscopy (STS)^31^ and scanning probe potentiometry (SPP)^47,48^ since it does not require a fixed sample-tip height or a flat surface and can be performed at a single constant tip and sample bias.

## Discussion

We have demonstrated the first simultaneous direct atomic scale STM and electrical operation on semiconductor nanowire devices. This is a significant step forward over previous SPM studies of biased nanostructures^32,33,49^ done at lower resolution, with a limited materials choice or on much larger thin film surfaces^19–21^. It is also an advance over our previous studies with combined AFM/STM measurements that we can remove surface oxide through atomic hydrogen cleaning without destroying the device, that we can scan on the nanowire without crashing onto an adjacent oxide layer and that STM offers considerably improved stability and resolution over AFM scanning on a curved nanowire surface. We combine all the properties needed for studies of the structural dynamics on nanoscale surfaces during device operation. This includes fast identification of a device and long term stable scanning even with voltage changes across the devices of several volts. Surface preparation can be performed with reactive gas and at elevated temperatures, a prerequisite for obtaining well defined surfaces. In contrast to our previous work, the method can be implemented in standard STM-only systems as well as low temperature systems without requiring additional *in*-*situ* microscopy tools such as SEM. Device fabrication use only commonly available processing technology.

Because InAs and GaAs have very different thermal, electrical and mechanical properties^50^, our high quality imaging on both materials (and on different crystal structures) indicate that our concepts should be applicable on many other nanostructured materials. The new device design also limits detrimental surface contamination of the wires due to processing steps as the nanowires can be deposited in the last step. Its fully conducting nature (when viewed from above) eliminates problems with charging and can therefore also be used in other important (electron based) microscopies such as surface sensitive photoelectron emission microscopy (PEEM)^51^ or SEM.

Our initial studies already reveal the interesting phenomena of device bias self-healing on InAs nanowire surfaces, as atomic scale defects were removed and morphology smoothed. This was an extremely surprising result since we would expect well known electromigration of In atoms in the material to produce and accumulate more defects and eventual structural failure of the nanowire. We observed no clear failure region nor did we observe an increase in electrical resistance associated with nanodevice failure, instead seeing reduced electrical resistance with defect removal and surface smoothing. We speculate that this is due to electric-field driven reordering (‘field annealing’) of the surface at below the threshold required for the nanowire to fail. Future measurements beyond the scope of the present proof-of-principle work will be needed to elucidate these effects in more detail.

While the importance of *in*-*situ* studies of semiconductor nanowires during growth has been clearly shown^52^, this demonstrates that surface structural change during operation can also be unusual and opens up possibilities for creating highly ordered crystalline surfaces with the use of bias voltages. Atomic scale structural changes due to induced voltages are central to devices that rely on defect diffusion and very small electric field induced structural changes for their function, such as memristors^53,54^. Our ability to rapidly switch voltages, and the small thermal effects can allow assessment of the impact of different suggested mechanism for structural change such as heating, electric current and pure voltage gradient effects^53^. Understanding structural dynamics will be central to go beyond trial-and-error in addressing the significant problems of performance degradation and failure over time found in many nanoscale structures.

## Methods

### Device manufacturing

Starting with n-type Si substrate HF etched free of thermally grown oxide both front and backside, we patterned by EBL in ma-n2403 negative resist strips of similar width as the length of the nanowires to be studied (and significantly shorter than the total length of the nanowire). We then deposited 60 nm SiO or SiO_2_ followed by a thin 15 nm Ti layer and 2 nm Au capping layer onto this pattern, leaving a trench in this material after lift-off and the Ti/Au layers on each side of it electrically separated from each other (typical resistance >5 MΩ) and the substrate. We choose to construct in this manner rather than the alternative of etching a trench into Ti-covered SiO_2_ on Si due to a) the greater control, consistency and resolution the negative resist EBL patterning afforded over wet etching, b) to prevent sidewall etching that could undercut the metal electrode under the nanowire and c) due to the preference to use Ti as the contact metal (also etched by standard SiO_2_ HF-based wet etching). We also wished to ensure perfectly conducting Si at the bottom of the trench with no patches of remaining residual oxide. We found the 2 nm Au cap under the nanowire to be sufficiently thin so as to not destroy the nanowire by alloying effects, whilst ensuring a highly conductive oxide-free surface on which to scan. We then deposited the nanowires by mechanical transfer, leaving a small number suspended between the Ti/Au electrodes. A second EBL step could be used to pattern a Ti ‘holding’ layer onto the nanowire on each side of the trench with thickness of 15 nm. The weak mechanical grip provided by the Ti holding layer was designed to ensure that the STM tip could not pull the nanowire from the metal surface whilst not inducing strain in the nanowire on heating. The thickness of this layer was empirically determined by thermally cycling a series of test devices at different layer thickness.

### Device preparation for STM

External electrical connection to the device was made by wire bonding to the Ti layer each side of the trench, with the rear of the sample grounded or optionally connected to a gate bias. Markers were patterned to allow the approximate initial positioning of the STM tip near a specific nanowire of interest and subsequent location of the relevant nanowire device without use of additional microscopy such as in vacuum Scanning Electron Microscopy (SEM). When bonded and mounted on a conducting sample plate the result is a fully conductive surface for the STM tip. This permits scanning anywhere on the sample surface whilst electrically isolating the nanowires from ground and allowing a bias to be applied only across them. The method retains the standard fabrication techniques and materials (Si, Ti, Au) in order to provide a realistic alternative that can be easily fabricated and keep many of the properties as the conventional lateral design.

### Nanowire location procedure

Using images taken elsewhere by optical microscope for navigation and identification of our position of the surface using the markers patterned on the surface by lithographic processing, we approximately positioned the STM tip using the standard tip-sample coarse positioning telescope camera (resolution 50 *μ*m). This could locate the tip within 100 *μ*m of the nanowire device. By moving in the direction of travel towards the trench, taking 10 *μ*m length STM scans in this direction, we could locate it’s position and then follow it’s edge to navigate to the relevant nanowire. This procedure required between 30 minutes and 1 hour to locate the desired nanowire within the STM scan window. Through further refinement and reducing the scan size, we then positioned the tip onto the nanowire on top of the contact, after which we could move freely along it’s length.

### Device in-vacuum cleaning and STM imaging

To enable STM experiments on crystalline clean nanowire surfaces, we removed the native oxide from the nanowires by exposing the full device to atomic hydrogen (H*) from a W filament thermal cracker at 1700 C for periods of 20 min at 350–450 C at chamber hydrogen pressure 1 × 10^−6^ mbar as described in detail elsewhere^27^. All preparation and STM imaging was performed in the same commercial Omicron system with ultra-high vacuum chamber (base pressure 1 × 10^−10^ mbar). We used standard tungsten wire STM tips, cleaned before imaging by Ar^+^ sputtering at 5 × 10^−6^ mbar chamber pressure of Ar. All images shown were recorded at negative sample voltages (filled state imaging) in the range between −1 to −2 V and with currents 0.05–0.2 nA. Cleaning was performed with the device sample mounted and electrically connected by wire bonding onto a sample plate with up to four Au electrical contacts that could be accessed from outside the vacuum chamber while the sample was in the STM. No indications of damage could be observed after cleaning in the STM system or subsequent *ex*-*situ* SEM images. We note a side effect from the cleaning process was to considerably enhance the conductance of the nanowire device (by several orders of magnitude) compared to the as-fabricated version prior to cleaning in air. This is shown in the I–V characteristics of an example device given in Supplementary Information Fig. 6.

## Electronic supplementary material

Supplementary Information
