# Supplementary material for: Imaging Atomic Scale Dynamics on III–V Nanowire Surfaces During Electrical Operation
Source: Sci Rep. 2017 Oct 6;7:12790. doi: 10.1038/s41598-017-13007-w (PMC5630597; doi:10.1038/s41598-017-13007-w)
Supplement: Supplementary file 1 — Supplementary Information [file 41598_2017_13007_MOESM1_ESM.pdf]

Imaging Atomic Scale Dynamics on III-V Nanowire Surfaces During Electrical Operation

J.L. Webb[1,*], J. Knutsson[1], M. Hjort[1], S.R. McKibbin[1], S. Lehmann[2], C. Thelander[2], K.A. Dick[2], R. Timm[1], and A. Mikkelsen[1]

[1]Division of Synchrotron Radiation Research, Lund University,Sweden
[2]Division of Solid State Physics, Lund University, Sweden
[*]james.webb@sljus.lu.se

ABSTRACT

As semiconductor electronics keep shrinking, functionality depends on individual atomic scale surface and interface features that may change as voltages are applied. In this work we demonstrate a novel device platform that allows scanning tunneling microscopy (STM) imaging with atomic scale resolution across a device simultaneously with full electrical operation. The platform presents a significant step forward as it allows STM to be performed everywhere on the device surface and high temperature processing in reactive gases of the complete device. We demonstrate the new method through proof of principle measurements on both InAs and GaAs nanowire devices with variable biases up to 4V. On InAs nanowires we observe a surprising removal of atomic defects and smoothing of the surface morphology under applied bias, in contrast to the expected increase in defects and electromigration-related failure. As we use only standard fabrication and scanning instrumentation our concept is widely applicable and opens up the possibility of fundamental investigations of device surface reliability as well as new electronic functionality based on restructuring during operation.

Supplementary Information

Here we detail the method for estimating the local surface potential V_L(x,y) from tip height during scanning on a typical nanodevice. The concept can be understood by displaying the height of the tip above the surface along a section of an InAs nanowire as V_b is varied as shown in Figure 1,a), where the height is plotted with respect to the tip height when the sample is fully grounded (in this case the ground level is slightly above the floating V_b=0 applied through the sourcemeter, accounting for the negative relative height on the figure at this value). We then plot this change in height (Δd) averaged along the wire as a function of applied bias in Supplementary Figure 1,b). We find an exponential dependence ('Fit 1') that could be expected from a change in local potential whilst maintaining a fixed tunnel current set-point and fixed V_{tip}. A more thorough analysis of this situation has been done in the work of Pronschinske et al.[1]. This gives a more precise theoretical calculation relating tip height change with V in constant current mode, with reasonable estimates for key parameters in their calculation for our device (work function=5.5eV, w=0.1, I=10pA, E_0=2.5eV using variables defined in the cited work). This functional form of Δd(V) arises as eV_t approaches the surface work function energy through a linear combination V_t=V_{tip}+V_b of the tip bias (V_{tip}=1.7V) with the applied bias (V_b=0-4V). The result of this calculation is shown as 'Fit 2' on Figure 1,b) with good agreement with the experimental data. These fits can then be used to give a direct quantitative relation between the height changes Δd and the local voltage potential V_L(x,y). Using the obtained direct relation between the height change and the local potential induced by the bias along the nanowire we estimate the potential drop across a 20nm section of the InAs nanowire device as approximately 0.04V for V_b=-4V. If a -4V applied potential dropped uniformly across the 1500±200nm of the exposed nanowire, this would give approximately 0.053V±0.006V over a 20nm length section. This assumes potential is dropped uniformly - in practice the drop may be higher at the contacts or over other defect features,which agrees well with the voltage drop observed experimentally here.

To identify changes in defect density due to the application of a bias through the nanowires a series of STM images were recorded before and after the application of a bias. A series of images would consists of approximately 10 images before and after. In Supplementary Figure 2 we show a before and after image zoomed in on the large terrace seen in Figure 3 of the main manuscript and with the greyscale contrast spread only over the intensities of the main terrace (thus saturating the intensities of the small island). We kept the island in the image as well as the stacking fault going through the image as they can be used to overlay the two images rather exactly with each other even if some drift has occurred between images. From the markings it can be seen that a majority of the adsorbates indicated by orange circles have disappeared, with the three largest having moved or changed otherwise. For the vacancies (indicated by blue squares) about half has disappeared while some have changed

otherwise and some are visible as vacancies still. We note again that no such changes could be observed in consecutive STM images before application of a bias through the nanowire. More vacancies and adsorbates can appear over time, but once they are present vacancies are not removed by scanning with the STM tip only and the bright protrusions also once stabilised are permanent.

Because we are imaging the surface over substantial time periods and tip changes might occur that potentially could obscure the observation of defects we have made a significant analysis of the images. In Supplementary Figure 3 we first show line profiles across and along the rows of atoms in the two images. From this we observe that the atomic resolution is in fact enhanced in the images recorded after a bias has been applied, where now the individual atoms in the rows can be identified. The resolution across the rows is fairly similar before and after.As for observing various adsorbates (large and small) before and after application of voltages we note that from Supplementary Figure 2 it can be seen that such adsorbates are easily identified as even the smallest ones protrude more than hundred pm beyond the atomic lattice.

Turning to the vacancies we show a section of the surface with two vacancies before application of voltages through the nanowires (dark hole in the atomic rows) and where one of the vacancies have disappeared after application of voltage but not the other. In the images on Supplementary Figure 4 it can be seen that the vacancy can be identified both before and after. Some of the accompanying variations of intensity on the atomic rows seen in the before picture cannot be seen in the after image. But the signature of the missing atom is observed.This is reasonable as the smaller changes in brightness are small variations in electronic structure, whereas the missing atom is a significant structural perturbation of the surface. If the charge state of a defect such as a vacancy changes this can also alter it appearance, but the signature of the missing atom will still be visible in the STM images (for more details on this point see for example ref 13 of the main manuscript).

Finally in Supplementary Figure 5 we show images of another session of measurements of STM imaging before and after where changes can be seen in larger scaler defects, even at very low biases applied through the wire.

Additionally in Supplementary Figure 6 we show an I-V through an InAs device, showing Ohmic conductance. We note that we observe a considerable increase (several order of magnitude) in conductance after treating an as-fabricated device with the atomic hydrogen cleaning process.

References

1. Pronschinske, A., Mardit, D. J. & Dougherty, D. B. Modeling the constant-current distance-voltage mode of scanning tunneling spectroscopy. *Phys. Rev. B* **84**, 205427 (2011).

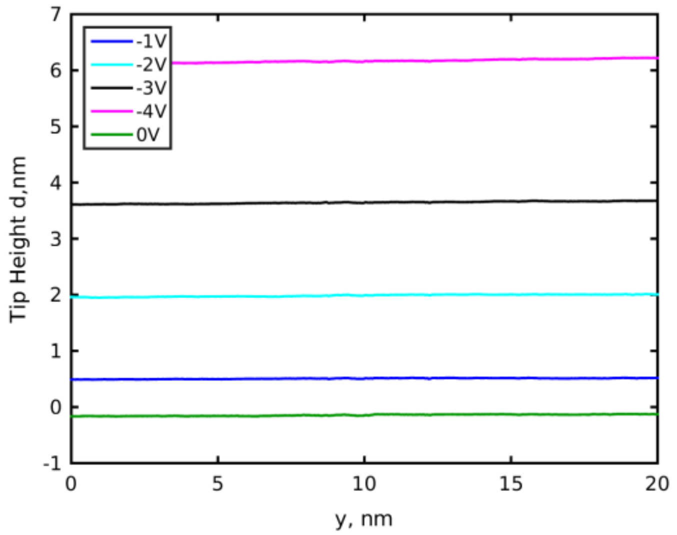

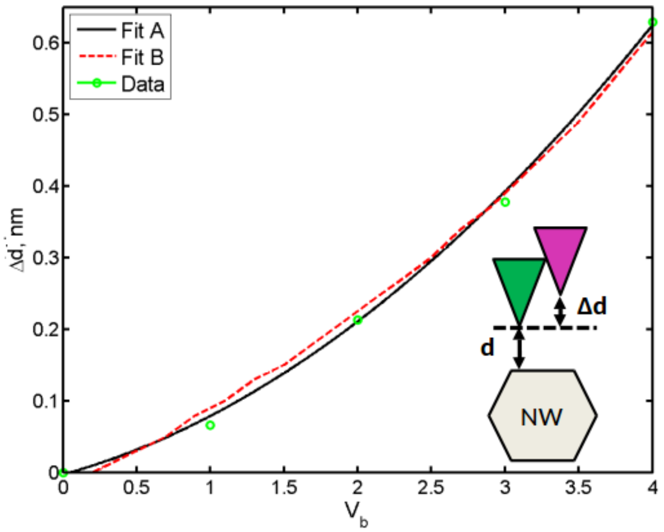

Supplementary Figure 1. a) The change in tip height d with respect to the device being fully grounded to the STM, scanning along length of an InAs nanowire device at V_b=0 to -4V. In this case the ground level is slightly above the floating V_b=0 applied through the sourcemeter, accounting for the negative relative height on the figure at this value. b) Average change in height Δd as a function of V_b, showing an exponential dependence between bias and tip height. Two fits are shown: Fit A to an exponential function and Fit B based on the work by Pronschinske et al.[1].

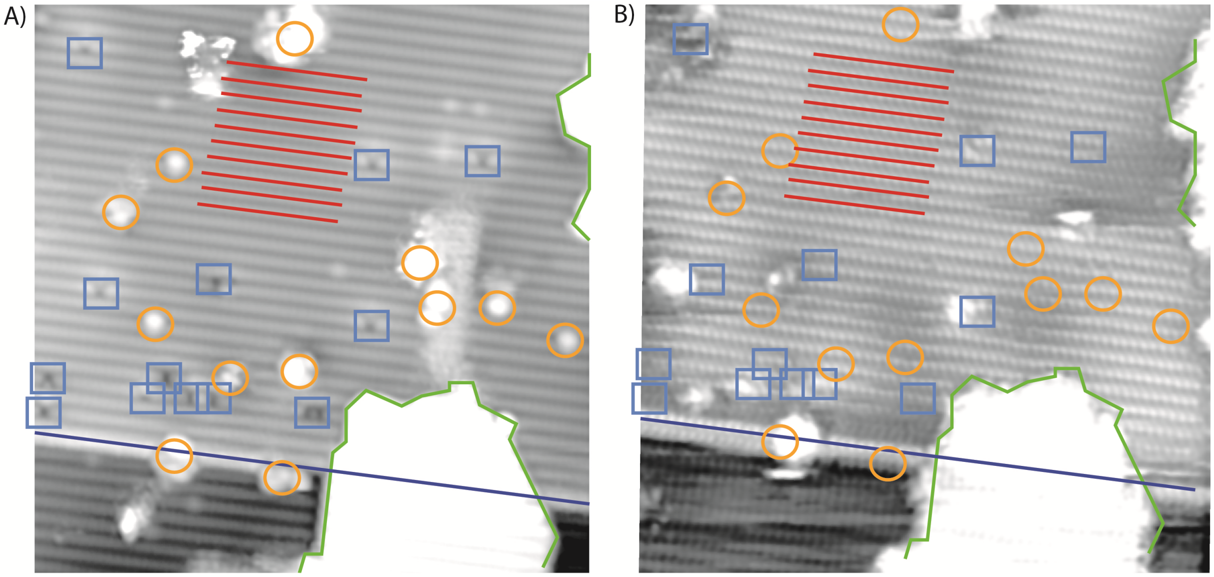

Supplementary Figure 2. A) STM image of a terrace on the InAs nanowire prior to voltages being applied through the NW. Vacancies in the surface are indicated by blue squares. Bright protrusions indicating adsorbed atoms or clusters of various sizes on top of the surface are indicated by orange circles. The position and presence of both the vacancies and the adsorbates does not change in several consecutive images before applying voltage through the NW as showing in Figure 3b) in the main text. An island found on the surface has been outlined using a green line and a stacking fault (and accompanying step) is indicated with a violet line. The atomic rows of the surface are indicated by red lines. B) STM image of a terrace on the InAs Nanowire after -2V has been applied to the NW. All the markers of defects identified on the image in A) has been exactly overlaid with the same positions in B) using the large island and the stacking fault. It can be seen that atomic rows can be equally well identified as in A). All STM images are recorded with a sample bias of -1.2V.

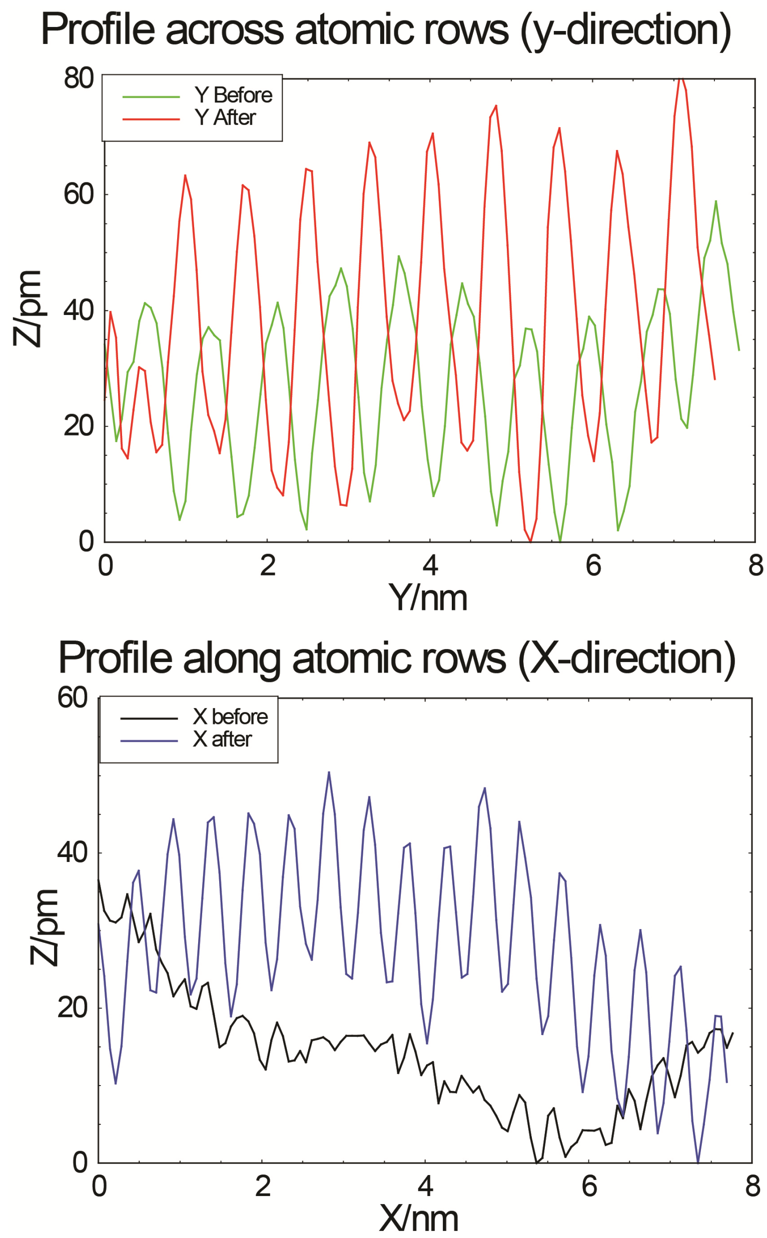

Supplementary Figure 3. Line profiles of the atomic rows from images (as in Supplementary Figure 2) across and along the rows from images recorded before (A) and after (B) application of bias

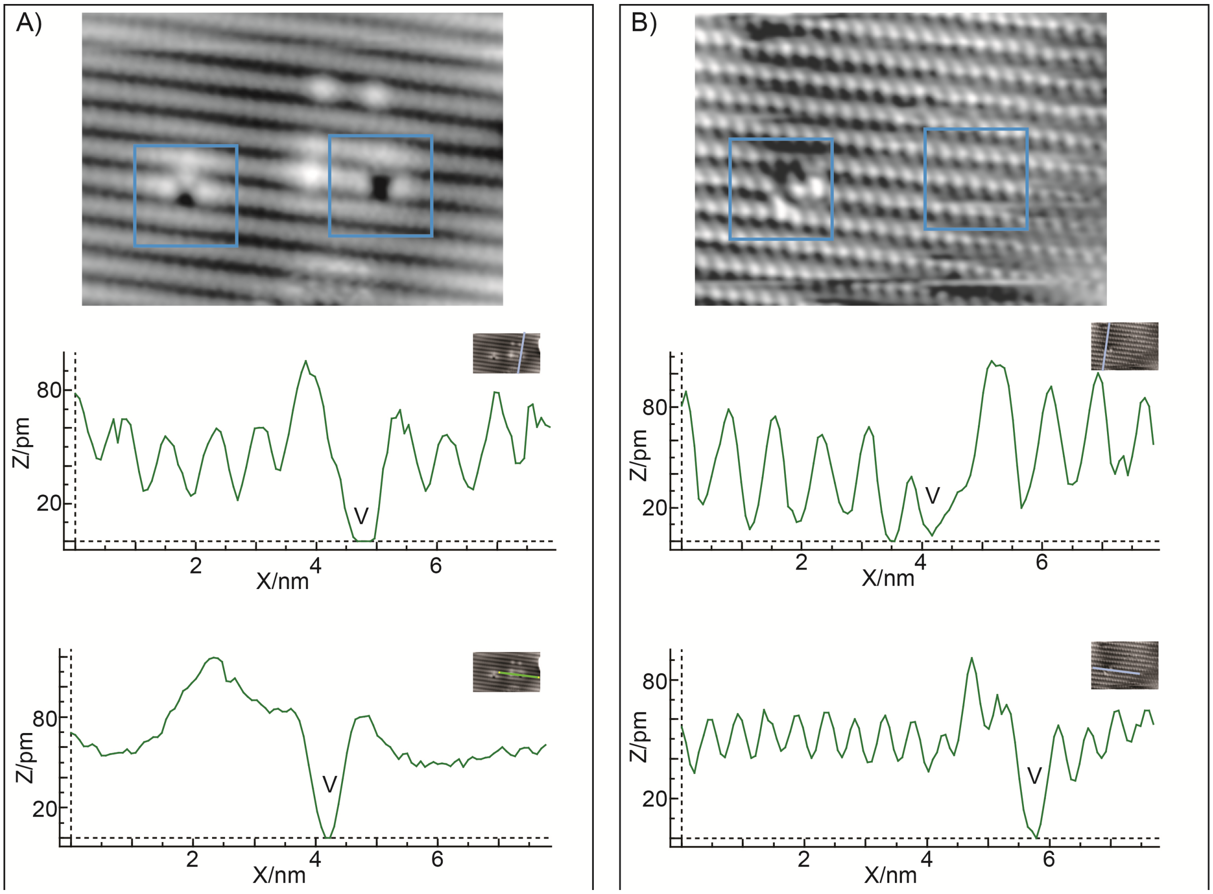

Supplementary Figure 4. A) STM image zoom in on two vacancies recorded prior to voltages being applied through the NW. Line profiles along and across one of the vacancies are shown below. B) STM image zoom in in exactly the same area recorded after voltages being applied through the NW. One of the vacancies has disappeared. Line profiles across and along the remaining vacancy are shown below.

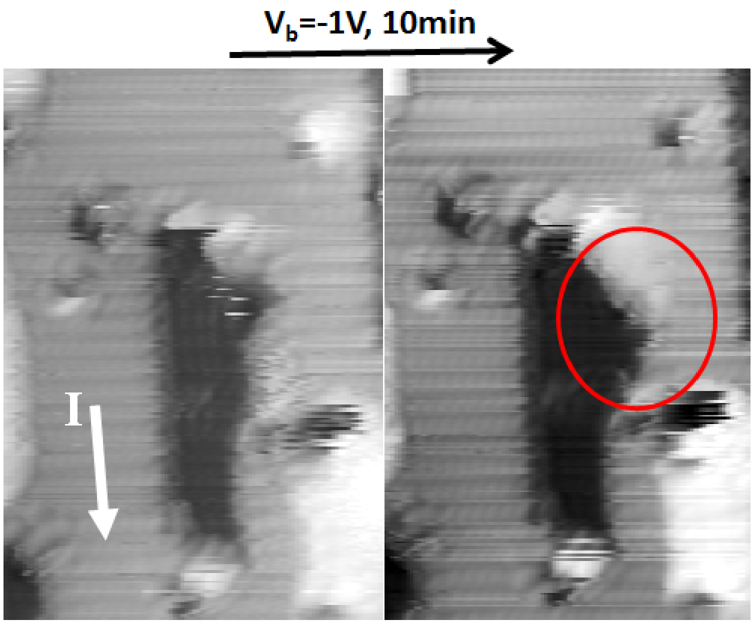

Supplementary Figure 5. Scan on a larger defect in a different area of the same nanowire device before (left) and after (right) the application of Vb=-1V. We observe the defect to change shape and reconstruct (circled) in the direction of current flow, indicated by a white arrow. Importantly, we observe no tip change between these two images. We consider -1V to be on the edge of the threshold required to move or eliminate vacancies - hence a number of surrounding defects remain unaltered.

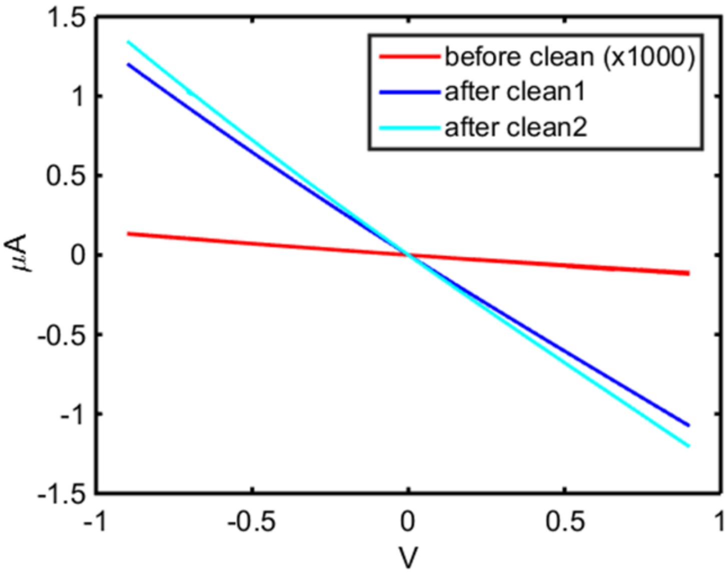

Supplementary Figure 6. I-V through an InAs device showing Ohmic conductance. We show the I-V before atomic hydrogen cleaning in UHV, after a first cleaning and after a subsequent second cleaning. We see the conductance typically improve by several orders of magnitude through this processing, which we attribute to the formation of a high quality, very low resistance junction between the electrode and the nanowire (possibly through Au/In alloying at the 300C+ high temperatures used) as a result of the cleaning process.
